# Multidisciplinary intensive rehabilitation treatment improves sleep quality in Parkinson’s disease

**DOI:** 10.1186/s40734-015-0020-9

**Published:** 2015-04-02

**Authors:** Giuseppe Frazzitta, Roberto Maestri, Davide Ferrazzoli, Giulio Riboldazzi, Rossana Bera, Cecilia Fontanesi, Roger P Rossi, Gianni Pezzoli, Maria F Ghilardi

**Affiliations:** Department of Parkinson Disease Rehabilitation, Moriggia-Pelascini Hospital, Gravedona ed Uniti, Fondazione Europea Ricerca Biomedica FERB, “S.Isidoro” Hospital, Trescore Balneario, Italy; Department of Biomedical Engineering, Scientific Institute of Montescano, S. Maugeri Foundation IRCCS, Montescano, Italy; Center for Parkinson’s Disease, Macchi Foundation, Varese and Department of Rehabilitation, “Le Terrazze” Hospital, Cunardo, Italy; Department of Physiol. Pharmacol. & Neuroscience, CUNY Medical School, Harris Hall 08, CCNY, 160 Convent Ave, New York, NY 10031 USA; The Graduate Center, Biology - Neuroscience PhD Program, CUNY, New York, NY USA; Department of Physical Medicine & Rehabilitation, JFK Johnson Rehabilitation Institute, Edison, NJ USA; NYU Movement Disorder Center, New York University, New York, NY USA; Parkinson Institute, Istituti Clinici di Perfezionamento, Milano, Italy; Department of Parkinson Rehabilitation, Ospedale Moriggia Pelascini, Via Pelascini 3, Gravedona ed Uniti, Como, 22015 Italy

**Keywords:** PDSS, Rehabilitation, Plasticity, Sleep quality

## Abstract

**Background:**

Sleep disturbances are among the most common non-motor symptoms of Parkinson’s disease (PD), greatly interfering with daily activities and diminishing life quality. Pharmacological treatments have not been satisfactory because of side effects and interactions with anti-parkinsonian drugs. While studies have shown that regular exercise improves sleep quality in normal aging, there is no definitive evidence in PD.

**Methods:**

In a retrospective study, we determined whether an intense physical and multidisciplinary exercise program improves sleep quality in a large group of patients with PD.

We analyzed the scores of PD Sleep Scale (PDSS), which was administered twice, 28 days apart, to two groups of patients with PD of comparable age, gender, disease duration and pharmacological treatment. The control group (49 patients) did not receive rehabilitation, The treated group (89 patients) underwent a 28-day multidisciplinary intensive rehabilitation program (three one-hour daily sessions comprising cardiovascular warm-up, relaxation, muscle-stretching, balance and gait training, occupational therapy to improve daily living activities).

**Results:**

At enrolment, control and treated groups had similar UPDRS and PDSS scores. At re-test, 28 days later, UPDRS and total PDSS scores improved in the treated (p < 0.0001) but not in the control group. In particular, the treated group showed significant improvement in PDSS scores for sleep quality, motor symptoms and daytime somnolence. The control group did not show improvement for any item.

**Conclusions:**

These results suggest that multidisciplinary intensive rehabilitation treatment may have a positive impact on many aspects of sleep in PD.

## Background

Sleep disturbances are among the most common non-motor symptoms of Parkinson’s disease (PD). Their prevalence ranges from 40% to 90%, depending upon the type of study, and they include insomnia, excessive daytime sleepiness, REM sleep behavior disorder, restless legs and sleep apnea [1,2]. Sleep problems greatly interfere with daily activities and diminish the quality of life of the patients and their caregivers. Unfortunately, these symptoms can be associated with the use of dopaminergic medications [3], and the pharmacological treatments have not produced satisfactory results [4,5]. Similarly, alternative approaches, such as repetitive transcranial magnetic stimulation, have been proven ineffectual [6].

In physiological aging, sleep complaints have been addressed mostly pharmacologically, but also with alternative or complementary strategies such as exercise: studies in normally aging subjects have indeed shown that sleep quality improves following regular exercises of moderate intensity [7,8]. There are increasing evidence that physical exercise of different sort and intensity can produce clinical benefits and might improve the general life quality of patients with PD [9,10]. Only few studies show a possible benefit of physical exercise on sleep in PD. *Nascimento et al.* demonstrated that a multimodal exercise program seem to be a feasible and effective alternative to decrease sleep-related disorders in people with PD and Alzheimer’s disease [11]. However, this exercise program is not specific for PD, no objective measures (i.e. physical performance test) were included, and the used scale for the assessment of sleep-related disorder (Mini-Sleep Questionnarie) [12] is not properly suitable for PD. Recently, *Wassom DJ et al.* determined the impact of a six-week Qigong exercise intervention as a potential complementary therapy in the management of sleep-related symptoms in PD. Following Qigong, subjects showed improvement in some aspects of sleep quality [13]. However this is a very small (seven patients) and uncontrolled study. Other promising indications come from a study showing that scores improved in several areas related to quality of life, including sleep, following a twelve-week exercise program [14]. However, also this study was uncontrolled and, most importantly, it used a non-PD specific scale (the Nottingham Health Profile) [15], in which sleep quality, one of six items, was tested with questions that did not address the specific problems of PD.

PD Sleep Scale (PDSS) [16] is a visual analogue scale with 15 questions related to sleep problems that are commonly associated with PD, such as REM behavior disorders and nocturnal motor symptoms. In the last ten years, PDSS has been developed, tested and used in patients with PD in different types of studies [6,17,18]. Importantly, PDSS has good test–retest reliability and it has been validated in different populations of PD patients, including an Italian population [17].

It has been demonstrated that the physical treatments specifically made for PD should be multidisciplinary and have certain characteristics of intensity in order to be effective [9,10].

In this retrospective study, we examined PDSS scores to verify the effects of a four-week multidisciplinary intensive rehabilitation program on the sleep quality in a large group of patients with PD.

## Methods

### Patients

We retrospectively analyzed data from our prospectively collected database of patients with PD. We studied 138 patients (61 men, mean age ± SD: 69.1 ± 7.4 years) belonging to two groups: *Group 1*- patients who underwent multidisciplinary intensive rehabilitation treatment (MIRT, 89 patients); Group 2- patients who were kept on pharmacological therapy only (Controls, 49 patients).

All patients had a diagnosis of “clinically probable” idiopathic PD [19]; Hoehn-Yahr stage 2 or 3; Mini-Mental State Examination score > 26; subjective complaints of sleep disturbances; ability to walk without physical assistance; ability to perceive visual and auditory cues. Also, they did not have any other neurological conditions, postural hypotension, cardiovascular disorders, musculoskeletal disorders, vestibular dysfunction limiting locomotion or balance. All patients had scores less than 8 at the Hamilton Depression scale.

This is a retrospective study based on our prospectively collected institutional database.

The Local Ethics Committee of Ospedale Moriggia-Pelascini approved the study. All patients provided written consent to the scientific treatment of their data in an anonymous form at the time of the assessment.

### MIRT protocol

MIRT had been described in detail in previous papers [10,20-22]. It is specifically designed for PD. Briefly, it consisted of a 4-week physical therapy that entailed three daily sessions, five days a week, in a hospital setting. The duration of each session, including recovery periods, was about one hour. The first session comprised cardiovascular warm-up activities, relaxation, muscle-stretching (scapular, hip flexor, hamstring and gastrocnemius muscles), exercises to improve the range of motion of spinal, pelvic and scapular joints, exercises to improve the functionality of the abdominal muscles, and postural changes in the supine position. The second session included exercises to improve balance and gait using a stabilometric platform with visual cues (patients had to follow a circular pathway on a screen by using a cursor sensitive to their feet movements on the platform) and treadmill plus (treadmill training with both visual and auditory cues) [23]. All the exercise on the treadmill were aerobic with a hearth rate reserve 60% to 70% and a maximum speed of treadmill scrolling of 3.5 km/h. The last was a session of occupational therapy to improve autonomy in day living activities: transferring from sitting to standing, rolling from supine to sitting position and viceversa, dressing, use of tools, exercises to improve hand functionality and visuo-motor skills. The rehabilitation program, considering individually each patient, could also include: speech therapy, hydrotherapy (for severe disorders of balance and posture), and robotic-assisted walking training for specific gait disorders (i.e. freezing of gait).

### Outcome measures

In both groups, we analyzed UPDRS III and II scores measured at enrolment and 28 days later, always at 10 AM, by the same neurologist, expert in movement disorders and blind with respect to the study design. Although UPDRS is a scale with some limitations (i.e. the low emphasis on the non-motor features of PD), we used this scale to establish the effect of MIRT on activities of daily life and on motor clinical aspects of the disease. These data were important in order to evaluate the correlations between the motor performance and the sleep parameters. Sleep complaints were assessed with an Italian translation of the PDSS [17]. Chaudhuri et al. showed that the PDSS is easy to use and is a reliable instrument for measuring sleep disturbances in PD [16]. Each of the items or questions was scored on a scale between 0 (always suffer from the disorder) and 10 (never suffers the disorder). The PDSS items were also grouped into sub-domains or categories of sleep-related disorders; sleep quality (items 1–3), nocturnal restlessness (item 4 and 5), nocturnal distressing dreams and hallucinations (item 6 and 7), nocturia (item 8), nocturnal motor off and sensory symptoms (items 9–13), and daytime somnolence (item 14 and 15). Classification of patients with nocturnal symptoms was based on a pre-specified cutoff score of less than or equal to 100; single items with a score below 8 were considered as “sleep disturbances” [16,24].

### Statistical analysis

Descriptive statistics are reported as mean ± SD. Shapiro–Wilk statistic was used to assess the normality of the distribution of all variables. To ascertain whether MIRT improved sleep quality, we used a mixed model ANOVA with Group (MIRT; Control) as between factor and Time (baseline, 28 days later) as within factor, and post-hoc tests. Correlations between PDSS, clinical and demographic data were assessed by the Pearson R coefficient. A p-value < 0.05 was considered statistically significant.

## Results

### PDSS at enrolment: characteristics of sleep in PD

Analyses on the combined group of 138 patients (MIRT: 89; Controls: 49) showed that, at enrolment, disease duration was 9.3 years (±3.3, SD); levodopa equivalent medication 611.5 mg (±341.8), Hoehn & Yahr stage 2.61 (±0.48), UPDRS II scores 11.4 (±5.5) and UPDRS III scores 14.87 (±7.1). On average, the total score of PDSS was 109.03 (±23.77) with 32% of the patients (44 in total, 36 in group 1 and 8 in group 2) having a total PDSS less or equal to 100. Table 1 reports average scores and percentages of patients with abnormal scores (<8) for each PDSS item. In general, these results were similar to the ones reported previously in different PD studies [16-18,24], with item 8 (nocturia) having the lowest scores and the greatest percentage of patients with abnormal scores.

**Table 1:** **Average scores (±SD) and percentage of patients with abnormal scores (<8)**

**Item**	**Mean Score ± SD**	**Abnormal Score (%)**
1	6.5 ± 2.6	57.2
2	6.6 ± 3.3	50.7
3	6.0 ± 3.2	65.2
4	7.3 ± 3.3	37.7
5	7.3 ± 3.1	42.8
6	8.4 ± 2.4	21.7
7	9.6 ± 1.3	5.1
8	4.2 ± 3.1	83.3
9	7.1 ± 3.4	39.9
10	7.6 ± 2.7	40.6
11	7.2 ± 2.9	47.8
12	7.3 ± 3.3	37.0
13	8.0 ± 2.9	27.5
14	7.2 ± 3.0	42.8
15	8.7 ± 1.9	26.1

We then correlated the global PDSS scores with the clinical and demographic data. Briefly, PDSS scores showed significant inverse correlation with UPDRS II scores (r = −0.29, p = 0.0005) and with the amount of levodopa equivalent therapy (r = −0.24, p = 0.005). No significant correlation was found between PDSS scores and age, gender, disease duration and UPDRS III scores.

Between-group comparisons at enrolment revealed no significant differences for disease duration (9.1 ± 3.6 vs. 9.7 ± 2.7 years, p = 0.32); levodopa equivalent medication (581.1 ± 351.7 vs. 666.8 ± 319.3 mg, p = 0.2), Hoehn & Yahr stage (2.61 ± 0.52 vs. 2.61 ± 0.44, p = 0.999) and UPDRS III scores (14.33 ± 5.97 vs. 15.86 ± 8.76, p = 0.23).

**Figure 1:**
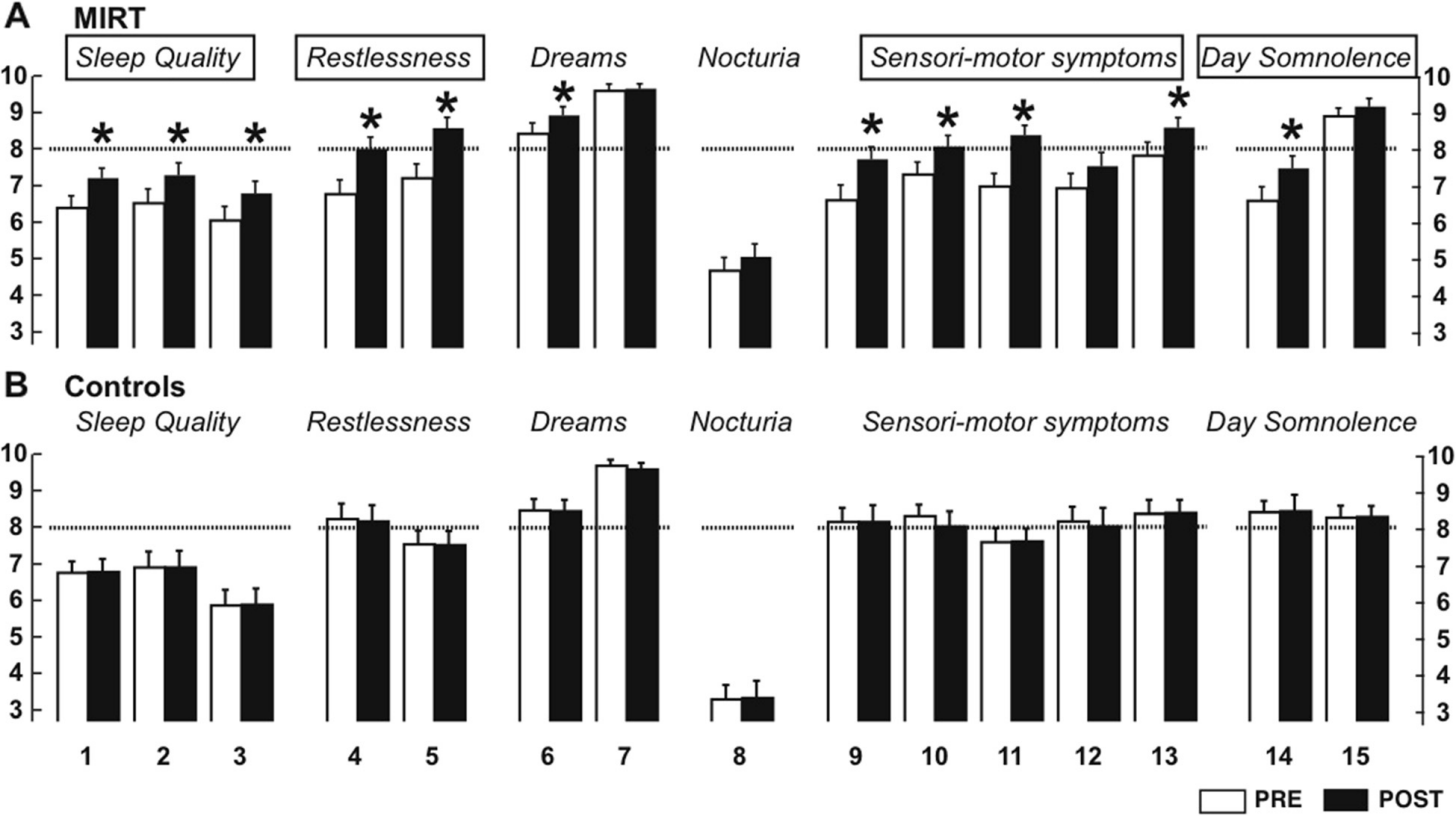
**Scores (mean ± SD) for PDSS items at baseline (white columns) and four weeks later (black columns). A**. *Multidisciplinary intensive rehabilitation treatment (MIRT) group;* **B**. *Control group.*Asterisks over columns and framed titles indicate significant score changes for single and grouped items, respectively. Thick dotted lines represent minimum normal scores.

The mean ± SD and the percentage of patients with abnormal scores (<8) for of each of the PDSS items are reported for each group in Figure 1 and Table 2. Briefly, at enrolment, there was no significant difference between the total scores (106.67 ± 27.29 vs. 113.3 ± 14.79, p = 0.12). However, we found between-group differences for some items: restlessness (item 4, MIRT < Controls, p = 0.013), nocturia (item 8, MIRT > Controls, p = 0.009), incontinence (item 9, MIRT < Controls, p = 0.01), numbness during sleep (item 10, MIRT < Controls, p = 0.04), pain at awakening (item 12, MIRT < Controls, p = 0.04), tiredness in the morning (item 14, MIRT < Controls, p = 0.0008), daytime sleepiness (item 15, MIRT > Controls, p = 0.05). Nevertheless, the scores’ profiles of the two groups were similar, with item 8 (nocturia) having the lowest scores and item 7 (hallucinations) the greatest scores (Figure 1).

**Table 2:** **Percentage of patients with abnormal scores (<8)**

	***Group 1***			***Group 2***		
**Item**	**PRE**	**POST**	**Difference**	**PRE**	**POST**	**Difference**
1	55.06	49.44	5.62	61.22	61.22	0
2	50.56	40.45	10.11	51.02	48.98	2.04
3	62.92	53.93	8.99	69.39	67.35	2.04
4	43.82	28.09	15.73	26.53	28.57	−2.04
5	39.33	22.47	16.85	48.98	48.98	0
6	22.47	20.22	2.25	20.41	20.41	0
7	5.62	6.74	−1.12	4.08	4.08	0
8	75.28	71.91	3.37	97.96	97.96	0
9	44.94	37.08	7.87	30.61	30.61	0
10	40.45	31.46	8.99	40.82	44.90	−4.08
11	47.19	22.47	**24.72**	48.98	48.98	0
12	40.45	31.46	8.99	30.61	32.65	−2.04
13	30.34	24.72	5.62	22.45	24.49	−2.04
14	51.69	35.96	15.73	26.53	26.53	0
15	20.22	13.48	6.74	36.73	36.73	0

### MIRT improves UPDRS scores and sleep quality

In both groups, pharmacological therapy did not change during the twenty-eight day period and it was the same at both testing times, thus ruling out a possible effects related to drug changes. Twenty-eight days after the baseline, UPDRS scores significantly decreased in the MIRT group (UPDRS III: 14.33 ± 5.97 vs. 8.34 ± 5.36 p < 0.0001; UPDRS II: 11.29 ± 4.88 vs. 6.63 ± 3.96, p < 0.0001), while they did not change in the controls (UPDRS III: 15.8 ± 8.76 vs. 15.65 ± 8.48, p = 0.23; UPDRS II: 11.69 ± 5.36 vs. 11.49 ± 6.44, p = 0.42).

An ANOVA on the total PDSS scores revealed a significant interaction between Groups and Time (Group: F(1,136) = 0.025, p = 0.88; Time: F(1,136) = 18.22; p < 0.0001; TimeXGroup: F(1,1) = 21.40; p < 0.0001), suggesting a significant change in the MIRT group only. On average, in the MIRT group, PDSS scores increased from 106.67 (±27.29) to 118.4 (±20.27; p < 0.0001), while they slightly decreased in the controls (from 113.3 ± 14.79 to 112.84 ± 14.78; p = 0.03). Also, as shown in Table 2 for each item, the percentage of patients with abnormal scores decreased in MIRT group and remained the same in the controls. In Figure 1, we report the scores of each item, separately and grouped by category, for the two groups at the two time points. Analysis of individual scores revealed that, in MIRT group, significant improvement occurred for almost all the items, with the exclusion of: items 7 (distressing hallucinations during the night) and 15 (daytime sleepiness), which had already average scores close to 10 before treatment; items 8 (nocturia) and 12 (painful posture in the morning), which showed only trends to improvement. In the controls, no statistical changes were noted, with the exception of a mild worsening for item 7 (from 9.63 ± 0.97 to 9.53 ± 1.0, p = 0.024). Thus, in summary, improvement in MIRT group was significant for the sensori-motor symptoms and restlessness (Group: F(1, 138) = 1.63, p = 0.2; Time: F(1, 138) = 4.14; p = 0.04; TimeXGroup: F(1,1) = 5.93; p = 0.0031; post-hoc: MIRT: p < 0.0001, controls: p = 0.24); the quality of sleep items (Group: F(1, 138) = 0.22, p = 0.64; Time: F(1, 138) = 9.17; p = 0.0029; TimeXGroup: F(1,1) = 8.49; p = 0.0042; post-hoc: MIRT: p < 0.0001, controls: p = 0.32); daytime somnolence (Group: F(1, 138) = 0.85, p = 0.36; Time: F(1, 138) = 4.3; p = 0.04; TimeXGroup: F(1,1) = 3.8; p = 0.049; post-hoc: MIRT: p < 0.0088; controls: p = 0.32).

We then verified whether the improvement in the general quality of sleep in the MIRT group was triggered by improvement in other categories or items: there were no significant correlations between improvements in any of the items or category and the quality of sleep. Nevertheless, we found significant correlations between improvements in nocturnal sensory-motor symptoms and daytime somnolence (r = 0.39; p = 0.001) as well as between restlessness (item 4 and 5) and distressing hallucinations during the night (r = 0.38; p = 0.001).

Finally, we found significant correlations in MIRT group between the changes in the PDSS total scores and in the UPDRS III and II scores (PDSS and UPDRS III: r = 0.26 p = 0.009; PDSS and UPDRS II: r = 0.35, p = 0.005). When we verified these correlations for each of the PDSS categories (Table 3), we found that sleep quality significantly correlated with the changes in UPDRS total and UPDRS II scores, as well as with the performance on the 6-minute walking time test. Improvement in the sensory-motor nocturnal symptoms instead significantly correlated with PDDS changes, while decrease in restlessness correlated with improvement in the total UPDRS scores.

**Table 3:** **Correlations between improvements in PDSS category and clinical scores (r values)**

	**Sleep quality**	**Day sleepiness**	**Motor & Sensory**	**Psychosis**	**Restlessness**
UPDRSTot	0.32*	0.08	0.20	0.05	0.29*
UPDRS_III	0.11	−0.07	0.01	−0.06	0.11
UPDRS_II	0.28*	0.07	0.14	0.07	0.21
PDDS	0.07	0.09	0.35*	0.10	0.21
sixMWT	−0.26*	0.04	−0.17	−0.16	−0.11

*Significant correlation between improvements in PDSS category and clinical scores (r values).

While the total scores and the PDSS general profiles were similar in the two groups at baseline, there were significant between-group differences for some of the PDSS items. Since the control group had greater scores closer to the normal range, although unlikely, over time improvement in this group could have been prevented by a ceiling effect. Thus, we selected a subgroup of controls with abnormal PDSS scores (<8) in at least one item at baseline and matched them with subjects of MIRT group by PDSS scores, UPDRS scores, age, and levodopa equivalents. This procedure left us with 30 subjects per group. ANOVAs comparing the effect of exercise and group revealed results similar to those obtained with the entire groups: for the total PDSS scores we found a significant interaction between Groups and Time (Group: F(1, 58) = 3.6, p = 0.064; Time: F(1, 58) = 36.3; p < 0.0001; TimeXGroup: F(1,1) = 41.8; p < 0.0001), showing again a significant score improvement in the MIRT group only. These results further suggest that absence of improvement in the control group is not due to a “ceiling” effect.

## Discussion and conclusions

The main result of this study is that a four-week intensive rehabilitation treatment in patients with PD has a positive effect on the sleep quality measured with PDSS, a scale that specifically addresses the sleep complaints of this disease. Second, the significant correlation between the improvements in UPDRS II and PDSS scores confirms that sleep plays an important role in improving autonomy in daily living activities and quality of life in patients with PD. Third, the finding of a significant inverse correlation between levodopa equivalents and PDSS scores suggests that increase of dopaminergic therapy has a negative impact on the quality of sleep of patients with PD. Altogether, these results suggest that sleep is an important factor in determining quality and autonomy in daily living activities in PD and that increasing levodopa levels might have a negative effect on sleep quality. Our data coincide with those recently appeared in recent studies showing that dopaminergic treatment decreases subjective and objective indices of sleep quality [25]. For instance, dopaminergic medication is thought to have a desynchronizing effect on sleep architecture that causes disruption of sleep continuity leading to excessive daytime sleepiness and “sleep attacks” [26]. Finally, these results confirm previous findings about the profile of the PDSS scores [15] in a different Italian population and suggest that PDSS yields highly reproducible results at one-month retest in the control group.

### MIRT has a positive effect on sleep quality and on nocturnal motor symptoms

As in previous studies [10,20,22], UPDRS II and III scores significantly decreased after MIRT, while they did not change in the control group after a similar time interval. The novel finding is that the scores of PDSS, a scale geared to measure sleep problems that are specific to PD, also improved following MIRT. The most significant effects were seen in the general quality of sleep, nocturnal motor symptoms and daytime somnolence.

A few papers have already reported that exercise has a positive effect on the general quality of sleep in elderly normal subjects [7,8]. This is the first study in a large PD population showing that, besides improving the general quality of sleep, exercise decreases the severity of the nocturnal symptoms that are typical of this disease. Although improvements in those two categories could be related, as the general quality of sleep might benefit from decreased severity of nocturnal parkinsonian symptoms, we did not find any significant correlation between them. Nocturnal motor and sensory-related symptoms such as restlessness, off periods, numbness, tingling and cramps, are characteristically found in a majority of patients with PD and are responsible for frequent arousals and disruption of sleep. The origins of the specific nocturnal symptoms as well as of problems falling asleep and maintaining sleep are still obscure, mostly because the effects of multiple factors with a possible causal role cannot be disentangled. In fact, on one side, the disease is accompanied by the degeneration of the brainstem regulatory centers of sleep and wakefulness; on the other, dopaminergic treatment contributes to exacerbating sleep problems. What could be the mechanisms behind this exercise-related improvement?

It is possible that the general improvement in several aspects of motor performance, measured with the UPDRS III scores, might lead to a reduction of nocturnal hypokinesia resulting in both an enhancement of sleep quality and a reduction of sensory-motor nocturnal symptoms. However, although nocturnal hypokinesia might be a major determinant of sleep quality [27], we did not find significant correlations between improvements in PDSS and UPDRS III scores. We instead found significant correlations between improvements in the scores of PDSS and UPDRS II, which reflect autonomy in daily living activities. Although we cannot establish direct causality between sleep improvement and quality of daytime life, undoubtedly, exercise improves the quality of both sleep and daytime life, with a significant gain in personal autonomy.

Therefore, we found that MIRT significantly improved the sleep quality of Parkinsonian patients. Although further studies are needed, these results strongly suggest that exercise should be included in the management of sleep disturbances in PD.

### The effects of MIRT on sleep might be linked to enhanced brain plasticity

A rather speculative interpretation of the mechanisms of MIRT effects on sleep quality might come from a recently formulated hypothesis, the synaptic homeostasis hypothesis [28], that provides a crucial link between plasticity-related phenomena and sleep. Briefly, this hypothesis states that during wake, activity induces long term potentiation (LTP)-related processes resulting in synaptic strength increases, which, in turn, promote sleep and, in particular, slow wave activity, the most restorative part of sleep. The function of slow wave activity is to renormalize, off-line, the synaptic weight and to restore the proper function, counteracting all costs of wake, both at cellular, system, and behavioral levels. Thus, more efficient are the LTP-related phenomena and the synapses strengthening during the day, greater is the slow wave activity during sleep. Indeed, exercise enhances the mechanisms related to brain plasticity, with positive effects on dopaminergic and glutamatergic neurotransmission [29], striatal upregulation of brain-derived neurotrophic factor (BDNF) and glial cell line-derived neurotrophic factor in rat models of PD [30], hippocampal neurogenesis in normal mice [31], increased levels of peripheral BDNF in the elderly [32] and in patients in the early PD stages [33].

In patients with PD, electrophysiological and behavioral induction of LTP-like phenomena in the cortex seems impaired, with decreased potentiation phenomena [34-36] and decreased retention of new skills [30,37,38]. In addition, slow wave activity during sleep is reduced in patients with PD [26]. We thus might speculate that exercise, by enhancing LTP-related phenomena and promoting plasticity during wakefulness, might trigger a more restorative sleep. Indeed, prospective studies are needed to verify this hypothesis and to determine whether exercise produces improvements in LTP-related mechanisms and whether this improvement induces, in turn, changes in the sleep electroencephalography.

### Study limitations

This study was based on retrospective analysis of a prospectically collected data-base. Even though this study design might constitute a potential limitation, we think that our results would not change significantly in a controlled study since data collection procedures were carefully designed and all relevant information were recorded on a structured protocol.

Although in this study was demonstrated a significant inverse correlation between levodopa equivalents and PDSS scores, the confounding effects of PD medications on sleep has not been completely addressed. Sleep disturbances in PD are dose related [39]: low-dose dopamine agonists have been associated with insomnia, whereas higher doses can lead to excessive daytime sleepiness. While initially associated with dopamine agonists, these symptoms can be induced by levodopa as well [3].

Limitations also include the different environmental conditions for the two groups. In fact, patients in the MIRT group were hospitalized for the entire 28-day period, while the controls were not. However, while hospitalization generally worsens sleep, in our study we have found an improving in sleep quality at the end of MIRT.

Another limitation arises from the difficulty to discern the effects related to the generic exercise and those directly attributable to MIRT. It is common opinion that better results in Parkinsonian patients have achieved using training program with a high training intensity, “beyond what they may self-select” [40], and with a multidisciplinary approach [41]. The aim of the study was to evaluate the efficacy of MIRT in Parkinsonian patients in comparison to patients treated only with drugs. The next step will be planning a study with a comparison between two different rehabilitation treatments.

A last criticism is the possible placebo effect of rehabilitation. The strength of expectation of clinical improvement during a rehabilitation or pharmacological treatment influences *per se* the release of dopamine [42]. This is another aspect that has to be clarified in a future study comparing two different rehabilitation treatments and providing for a follow-up period.

## Abbreviations

PD: Parkinson’s disease
PDSS: PD sleep scale
MIRT: Multidisciplinary intensive rehabilitation treatment
UPDRS: Unified Parkinson’s disease rating scale
LTP: Long term potentiation
BDNF: Brain-derived neurotrophic factor

## References

[1] Barone P, Antonini A, Colosimo C, Marconi R, Morgante L, Avarello TP (2009). The PRIAMO study: A multicenter assessment of nonmotor symptoms and their impact on quality of life in Parkinson’s disease. Mov Disord.

[2] Kumar S, Bhatia M, Behari M (2002). Sleep disorders in Parkinson’s disease. Mov Disord.

[3] Park A, Stacy M (2011). Dopamine-induced nonmotor symptoms of Parkinson’s disease. Parkinsons Dis.

[4] Budur K, Rodriguez C, Foldvary-Schaefer N (2007). Advances in treating insomnia. Cleve Clin J Med.

[5] Paus S, Brecht HM, Koster J, Seeger G, Klockgether T, Wullner U (2003). Sleep attacks, daytime sleepiness, and dopamine agonists in Parkinson’s disease. Mov Disord.

[6] Arias P, Vivas J, Grieve KL, Cudeiro J (2010). Double-blind, randomized, placebo controlled trial on the effect of 10 days low-frequency rTMS over the vertex on sleep in Parkinson’s disease. Sleep Med.

[7] King AC, Oman RF, Brassington GS, Bliwise DL, Haskell WL (1997). Moderate-intensity exercise and self-rated quality of sleep in older adults. A randomized controlled trial. JAMA.

[8] Santos RV, Viana VA, Boscolo RA, Marques VG, Santana MG, Lira FS (2012). Moderate exercise training modulates cytokine profile and sleep in elderly people. Cytokine.

[9] Ellis T, Katz DI, White DK, DePiero TJ, Hohler AD, Saint-Hilaire M (2008). Effectiveness of an inpatient multidisciplinary rehabilitation program for people with Parkinson disease. Phys Ther.

[10] Frazzitta G, Bertotti G, Riboldazzi G, Turla M, Uccellini D, Boveri N (2012). Effectiveness of intensive inpatient rehabilitation treatment on disease progression in parkinsonian patients: a randomized controlled trial with 1-year follow-up. Neurorehabil Neural Repair.

[11] Nascimento CM, Ayan C, Cancela JM, Gobbi LT, Gobbi S, Stella F (2014). Effect of a multimodal exercise program on sleep disturbances and instrumental activities of daily living performance on Parkinson’s and Alzheimer’s disease patients. Geriatr Gerontol Int.

[12] Gorenstein C (1983). Reliability of a sleep self-evaluation questionnaire. AMB Rev Assoc Med Bras.

[13] Wassom DJ, Lyons KE, Pahwa R, Liu W. Qigong exercise may improve sleep quality and gait performance in Parkinson’s disease: a pilot study. Int J Neurosci 2014, Oct 22. [Epub ahead of print]. doi:10.3109/00207454.2014.966820. 10.3109/00207454.2014.966820 25233147

[14] Rodrigues De Paula F, Teixeira Salmela LF, Coelho De Morais Faria CD, Rocha De Brito P, Cardoso F (2006). Impact of an exercise program on physical, emotional, and social aspects of quality of life of individuals with Parkinson’s disease. Mov Disord.

[15] Hunt SM, McKenna SP, McEwen J, Backett EM, Williams J, Papp E (1980). A quantitative approach to perceived health status: a validation study. J Epidemiol Community Health.

[16] Chaudhuri KR, Pal S, DiMarco A, Whately-Smith C, Bridgman K, Mathew R (2002). The Parkinson’s disease sleep scale: a new instrument for assessing sleep and nocturnal disability in Parkinson’s disease. J Neurol Neurosurg Psychiatry.

[17] Pellecchia MT, Antonini A, Bonuccelli U, Fabbrini G, Ferini Strambi L, Stocchi F (2012). Observational study of sleep-related disorders in Italian patients with Parkinson’s disease: usefulness of the Italian version of Parkinson’s disease sleep scale. Neurol Sci.

[18] Trenkwalder C, Kies B, Rudzinska M, Fine J, Nikl J, Honczarenko K (2011). Rotigotine effects on early morning motor function and sleep in Parkinson’s disease: a double-blind, randomized, placebo-controlled study (RECOVER). Mov Disord.

[19] Gelb DJ, Oliver E, Gilman S (1999). Diagnostic criteria for Parkinson disease. Arch Neurol.

[20] Frazzitta G, Morelli M, Bertotti G, Felicetti G, Pezzoli G, and Maestri R. Intensive Rehabilitation Treatment in Parkinsonian Patients with Dyskinesias: A Preliminary Study with 6-Month Follow-up. Parkinson’s Disease 2012, (2012). Article ID 910454, 4 pages. doi:10.1155/2012/910454. 10.1155/2012/910454 PMC3372063 22701812

[21] Frazzitta G, Bertotti G, Uccellini D, Boveri N, Rovescala R, Pezzoli G, Maestri R. Short- and Long-Term Efficacy of Intensive Rehabilitation Treatment on Balance and Gait in Parkinsonian Patients: A Preliminary Study with a 1-Year Follow-up. Parkinson’s Disease 2013, (2013) Article ID 583278, 5 pages. doi:10.1155/2013/583278. 10.1155/2013/583278 PMC3677635 23766927

[22] Frazzitta G, Maestri R, Bertotti G, Riboldazzi G, Boveri N, Perini M, Uccellini D, Turla M, Comi C, Pezzoli G, Ghilardi MF. Intensive Rehabilitation Treatment in Early Parkinson’s Disease: A Randomized Pilot Study With a 2-Year Follow-Up. Neurorehabilitation and Neural Repair 2014, 2014. 10.1177/1545968314542981 25038064

[23] Frazzitta G, Maestri R, Uccellini D, Bertotti G, Abelli P (2009). Rehabilitation treatment of gait in patients with Parkinson’s disease with freezing: a comparison between two physical therapy protocols using visual and auditory cues with or without treadmill training. Mov Disord.

[24] Chaudhuri RK, Martinez-Martin P, Rolfe KA, Cooper J, Rockett CB, Giorgi L (2012). Improvements in nocturnal symptoms with ropinirole prolonged release in patients with advanced Parkinson’s disease. Eur J Neurol.

[25] Chahine LM, Daley J, Horn S, Duda JE, Colcher A, Hurtig H (2013). Association between dopaminergic medications and nocturnal sleep in early-stage Parkinson’s disease. Parkinsonism Relat Disord.

[26] Brunner H, Wetter TC, Hogl B, Yassouridis A, Trenkwalder C, Friess E (2002). Microstructure of the non-rapid eye movement sleep electroencephalogram in patients with newly diagnosed Parkinson’s disease: effects of dopaminergic treatment. Mov Disord.

[27] Louter M, Munneke M, Bloem BR, Overeem S (2012). Nocturnal hypokinesia and sleep quality in Parkinson’s disease. J Am Geriatr Soc.

[28] Tononi G, Cirelli C (2014). Sleep and the price of plasticity: from synaptic and cellular homeostasis to memory consolidation and integration. Neuron.

[29] Petzinger GM, Fisher BE, Van Leeuwen JE, Vukovic M, Akopian G, Meshul CK (2010). Enhancing neuroplasticity in the basal ganglia: the role of exercise in Parkinson’s disease. Mov Disord.

[30] Cotman CW, Berchtold NC, Christie LA (2007). Exercise builds brain health: key roles of growth factor cascades and inflammation. Trends Neurosci.

[31] van Praag H, Shubert T, Zhao C, Gage FH (2005). Exercise enhances learning and hippocampal neurogenesis in aged mice. J Neurosci.

[32] Voss MW, Prakash RS, Erickson KI, Basak C, Chaddock L, Kim JS (2010). Plasticity of brain networks in a randomized intervention trial of exercise training in older adults. Front Aging Neurosci.

[33] Frazzitta G, Maestri R, Ghilardi MF, Riboldazzi G, Perini M, Bertotti G (2014). Intensive Rehabilitation Increases BDNF Serum Levels in Parkinsonian Patients: A Randomized Study. Neurorehabil Neural Repair.

[34] Kishore A, Joseph T, Velayudhan B, Popa T, Meunier S (2012). Early, severe and bilateral loss of LTP and LTD-like plasticity in motor cortex (M1) in de novo Parkinson’s disease. Clin Neurophysiol.

[35] Koch G (2013). Do studies on cortical plasticity provide a rationale for using Non-invasive brain stimulation as a treatment for Parkinson’s disease patients?. Front Neurol.

[36] Morgante F, Espay AJ, Gunraj C, Lang AE, Chen R (2006). Motor cortex plasticity in Parkinson’s disease and levodopa-induced dyskinesias. Brain.

[37] Bedard P, Sanes JN (2011). Basal ganglia-dependent processes in recalling learned visual-motor adaptations. Exp Brain Res.

[38] Marinelli L, Crupi D, Di Rocco A, Bove M, Eidelberg D, Abbruzzese G (2009). Learning and consolidation of visuo-motor adaptation in Parkinson’s disease. Parkinsonism Relat Disord.

[39] Verbaan D, van Rooden SM, Visser M, Marinus J, van Hilten JJ (2008). Nighttime sleep problems and daytime sleepiness in Parkinson’s disease. Mov Disord.

[40] Hirsch MA, Farley BG (2009). Exercise and neuroplasticity in persons living with Parkinson’s disease. Eur J Phys Rehabil Med.

[41] Post B, van der Eijk M, Munneke M, Bloem BR (2011). Multidisciplinary care for Parkinson’s disease: not if, but how!. Pract Neurol.

[42] Lidstone SC, Schulzer M, Dinelle K, Mak E, Sossi V, Ruth TJ (2010). Effects of expectation on placebo-induced dopamine release in Parkinson disease. Arch Gen Psychiatry.

